# Purpuric rash on the legs of a patient with coronavirus disease

**DOI:** 10.1590/0037-8682-0464-2020

**Published:** 2020-09-21

**Authors:** Diego Henrique Morais Silva, Agatha Ramos Oppenheimer, Thais do Amaral Carneiro Cunha

**Affiliations:** 1 Hospital do Servidor Público Estadual de São Paulo, Programa de Residência Médica em Dermatologia, São Paulo, SP, Brasil.; 2 Hospital do Servidor Público Estadual de São Paulo, Departamento de Dermatologia, São Paulo, SP, Brasil.

**FIGURE 1:**
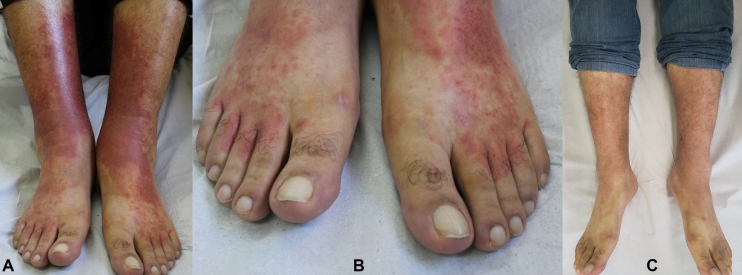
**(A):** Purpuric rashes on the anterior surface of the legs. **(B):** Characteristics of the lesions in the toes, resembling perniosis. (C): Clinical improvement after 2 weeks of expectant management.

A 30-year-old male health-care worker, who was regularly monitored at the dermatology clinic for ochre dermatitis on the medial side of the legs, reported a 15-day history of fever and diarrhea, followed by erythematous-purpuric macules and papules on the legs and feet 1 week before, involving some fingers (Figures 1A and B). He had no history of pain or itching. After 2 weeks, he returned to the clinic after testing for coronavirus disease (COVID-19) antibodies (IgM and IgG positive). The skin lesions showed considerable improvement during this period (Figure 1C) and the patient fully recovered at home.

The patient' clinical condition was considered a vascular skin manifestation associated with COVID-19 infection^1^ ^,^ ^2^. Among the specific dermatological findings of the disease, pernio-like lesions are highlighted^2^. Perniosis is a vascular inflammatory response occurring in the extremities and is clinically characterized by erythematous-purpuric macules and papules. This skin condition can be induced by low temperatures or can be associated with autoimmune diseases, drugs, or infections. When associated to COVID-19, pernio-like lesions are predominantly manifest in young and healthy patients, which may represent a good prognosis of the infectious condition^2^ ^,^ ^3^.

Severe acute respiratory syndrome coronavirus 2 infection can affect several organ systems, including the skin^1^. As Brazil is one of the most affected countries, recognizing associated skin lesions is important for the early diagnosis of COVID-19.
